# Correction: COVID-19 and flu: conserved or specific immune signature?

**DOI:** 10.1038/s41423-022-00914-w

**Published:** 2022-08-30

**Authors:** Christophe Paget, François Trottein

**Affiliations:** 1 Centre d’Etude des Pathologies Respiratoires, INSERM U1100, Université de Tours, Faculté de Médecine de Tours, 37000 Tours, France; 2 Centre d’Infection et d’Immunité de Lille, INSERM U1019, CNRS UMR 9017, Université de Lille, CHU Lille, UMS 2014 - PLBS, U1019, Institut Pasteur de Lille, 59000 Lille, France

**Keywords:** Predictive markers, Diagnostic markers

Correction to: *Cellular & Molecular Immunology* 10.1038/s41423-020-00595-3, published online 11 January 2021

The licence information was missing from this article and should have been CC-BY.

The original article has been corrected.

